# Supplementary material for: Ocean surface energy balance allows a constraint on the sensitivity of precipitation to global warming
Source: Nat Commun. 2021 Apr 9;12:2115. doi: 10.1038/s41467-021-22406-7 (PMC8035209; doi:10.1038/s41467-021-22406-7)
Supplement: Supplementary file 1 — Supplementary Information [file 41467_2021_22406_MOESM1_ESM.docx]

**Supplementary information**

**Ocean surface energy balance allows a constraint on the sensitivity of precipitation to global warming**

Wei Wang^1+^, TC Chakraborty^2+^, Wei Xiao^1^, Xuhui Lee^2*^

^1^Yale-NUIST Center on Atmospheric Environment, Nanjing University of Information Science & Technology, Nanjing 210044, China

^2^School of the Environment, Yale University, New Haven, CT 06511, USA.

^+^ Authors of equal contribution

^*^ Corresponding author: [xuhui.lee@yale.edu](mailto:xuhui.lee@yale.edu)

**Supplementary Figure 1.** **Ocean surface albedo as a function of global mean 2-m temperature.** The annual ocean surface albedo is the ratio of the area-weighted mean outgoing shortwave radiation to the incoming shortwave radiation observed by the Clouds and the Earth’s Radiation Energy System (CERES Edition 4.1, <https://ceres.larc.nasa.gov/data/>). Global mean temperature anomalies are from GISS Surface Temperature Analysis (GISTEMP v4,

<https://data.giss.nasa.gov/gistemp/>). The solid line represents linear regression with the regression statistics noted (*N*, number of years; *R*, linear correlation coefficient).

**Supplementary Figure 2.** **Relationship between the land modifier (*φ*) and tree fraction change**. The solid line represents linear regression with the regression statistics noted (*N*, number of experiments; *R*, linear correlation coefficient). Error bars are ± one standard deviation. Description of model scenarios is given in Figure 1 caption.


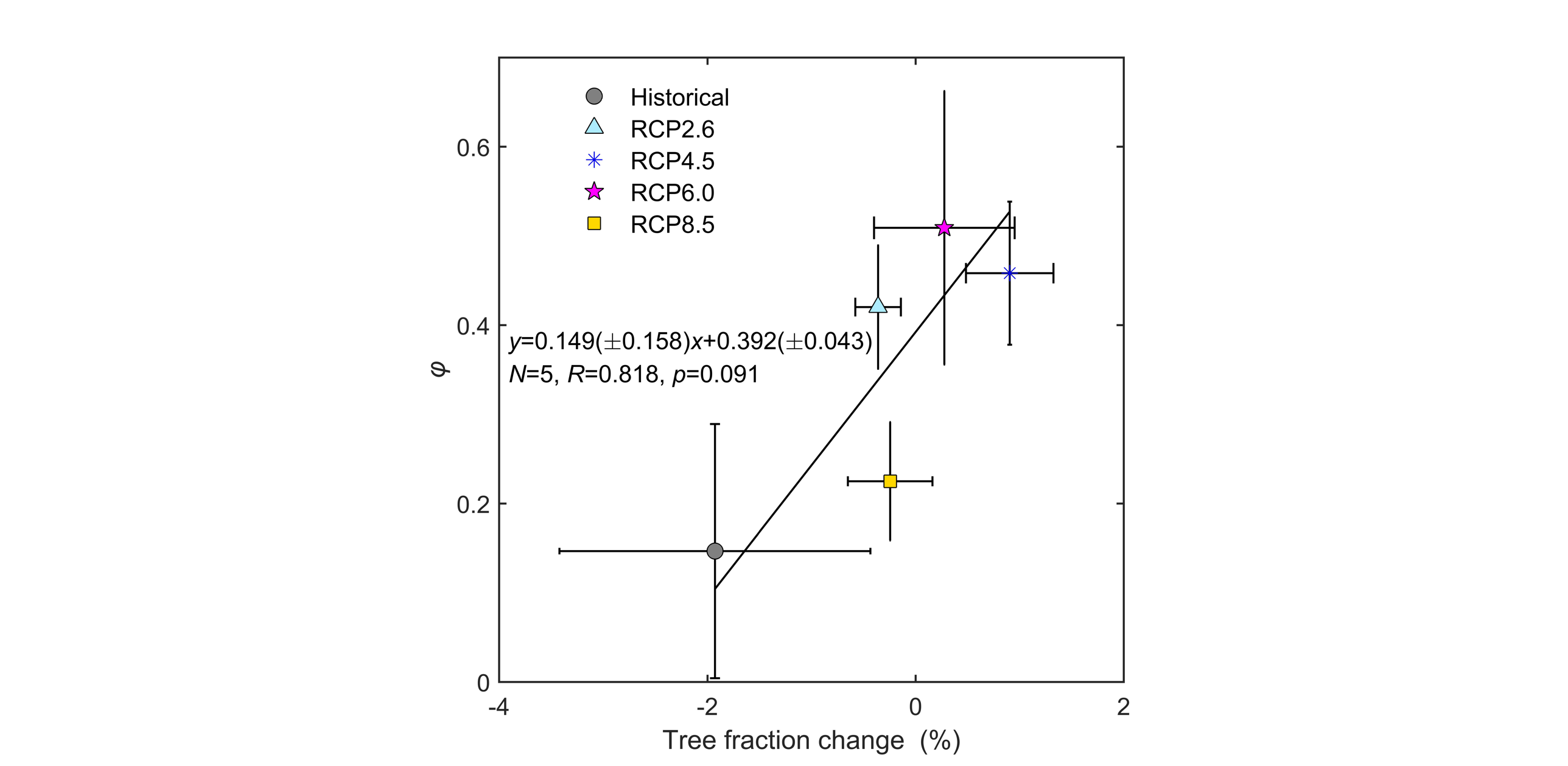


**Supplementary Figure 3. Components of the global ocean surface energy balance.** The flux is positive in the direction indicated and negative if it goes against the direction shown. *a* – albedo; *β* – Bowen ratio; net radiation $R_{n}=\left( 1-a \right)K_{\downarrow}+L_{\downarrow}-L_{\uparrow}$.

**
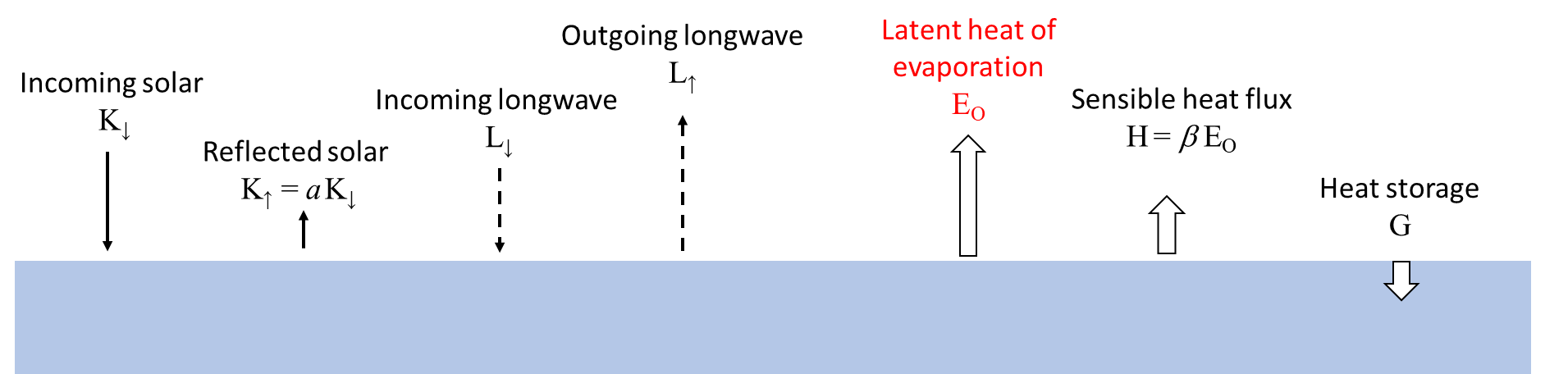
**

**Supplementary Figure 4. Interdependence between temperature sensitivities of incoming shortwave (Δ*K*_↓_/Δ*T*) and longwave radiation (Δ*L*_↓_/Δ*T*) at the ocean surface.** The solid line represents linear regression with the regression statistics noted (*N*, number of model experiments; *R*, linear correlation). One outlier, marked by light blue filled triangle with the model name noted, is excluded from the statistical calculation. The white circle with error bars (± 1 standard deviation) denotes the observational constraint. The white square denotes sensitivities due to atmospheric moistening under clear skies^1^. Description of model scenarios is given in Figure 1 caption.


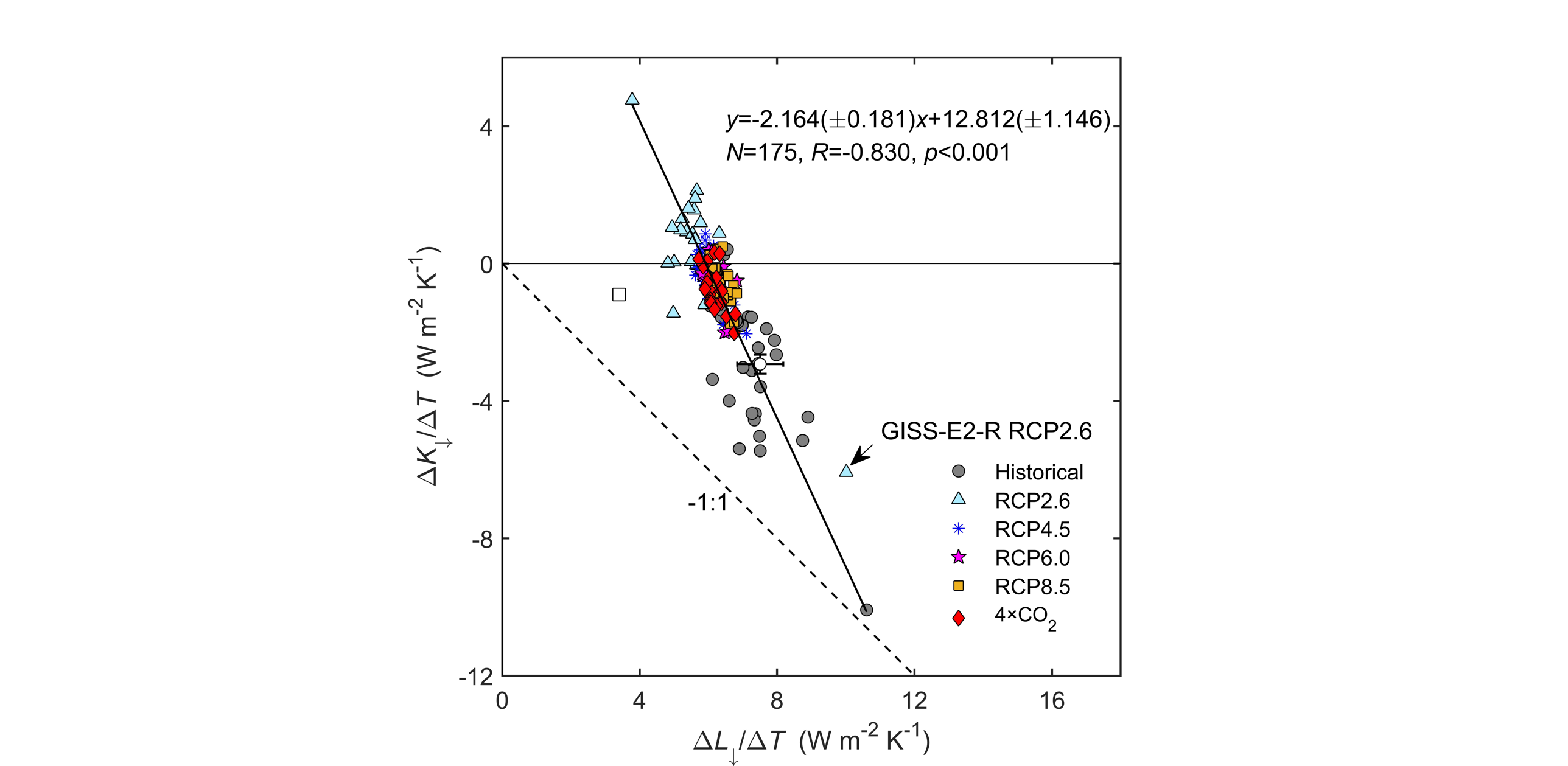


**Supplementary Figure 5. Comparison between CMIP5 RCP8.5 and CMIP6 ssp585 scenarios**. **a**, Component contributions to global precipitation temperature sensitivity (Δ*P*/Δ*T*). Error bars are ± one standard deviation. **b**, Relationship between changes in global precipitation ∆*P* and ocean evaporation ∆*E*_O_, with regression statistics indicated (*N*, number of model experiments; *R*, linear correlation). **c**, Emergent constraint on global precipitation temperature sensitivity, where the *x*-axis is the same as in Figure 4a. Solid line represents the regression equation in Figure 4a. Description of model scenarios is given in Figure 1 caption.


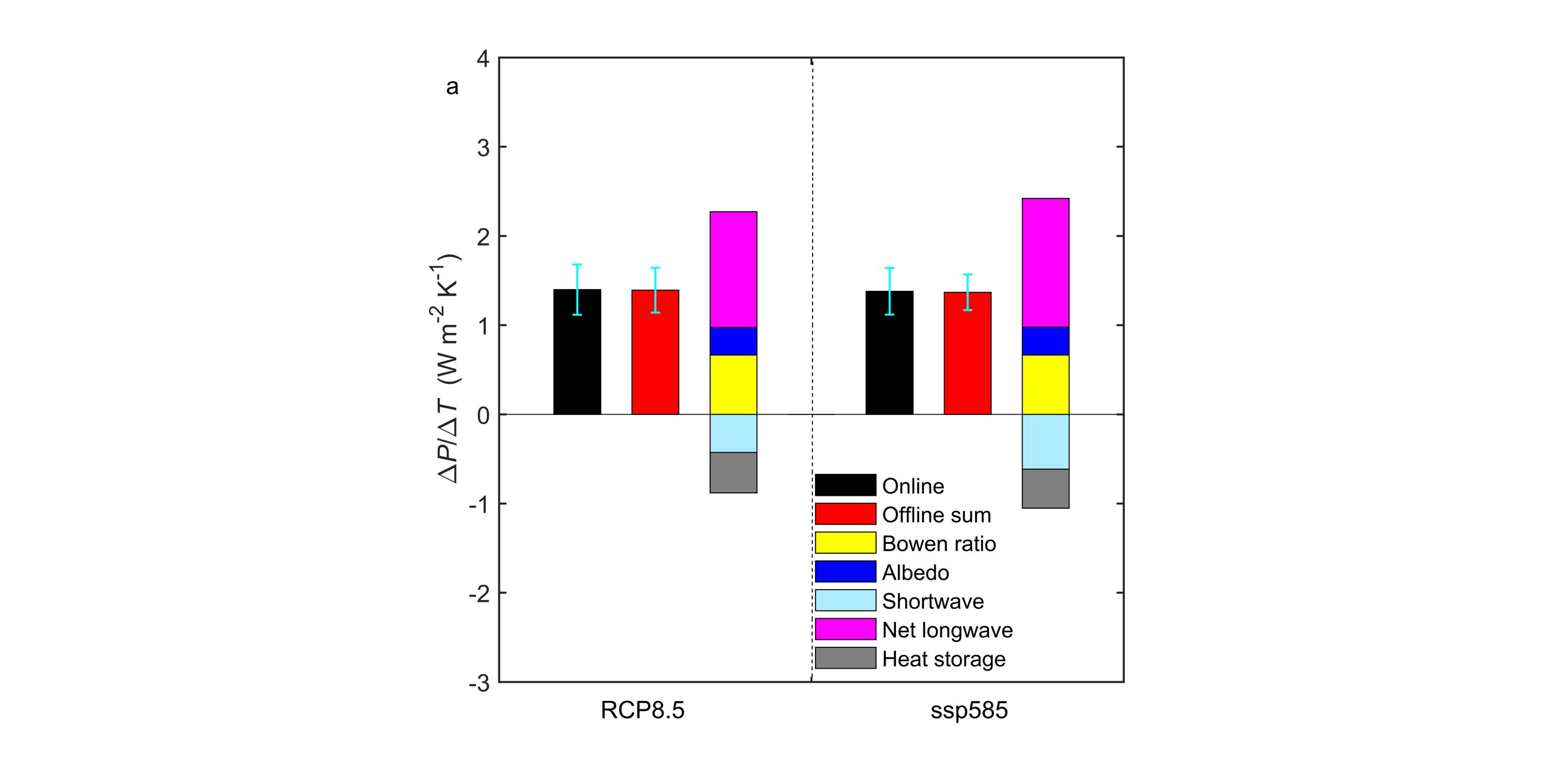

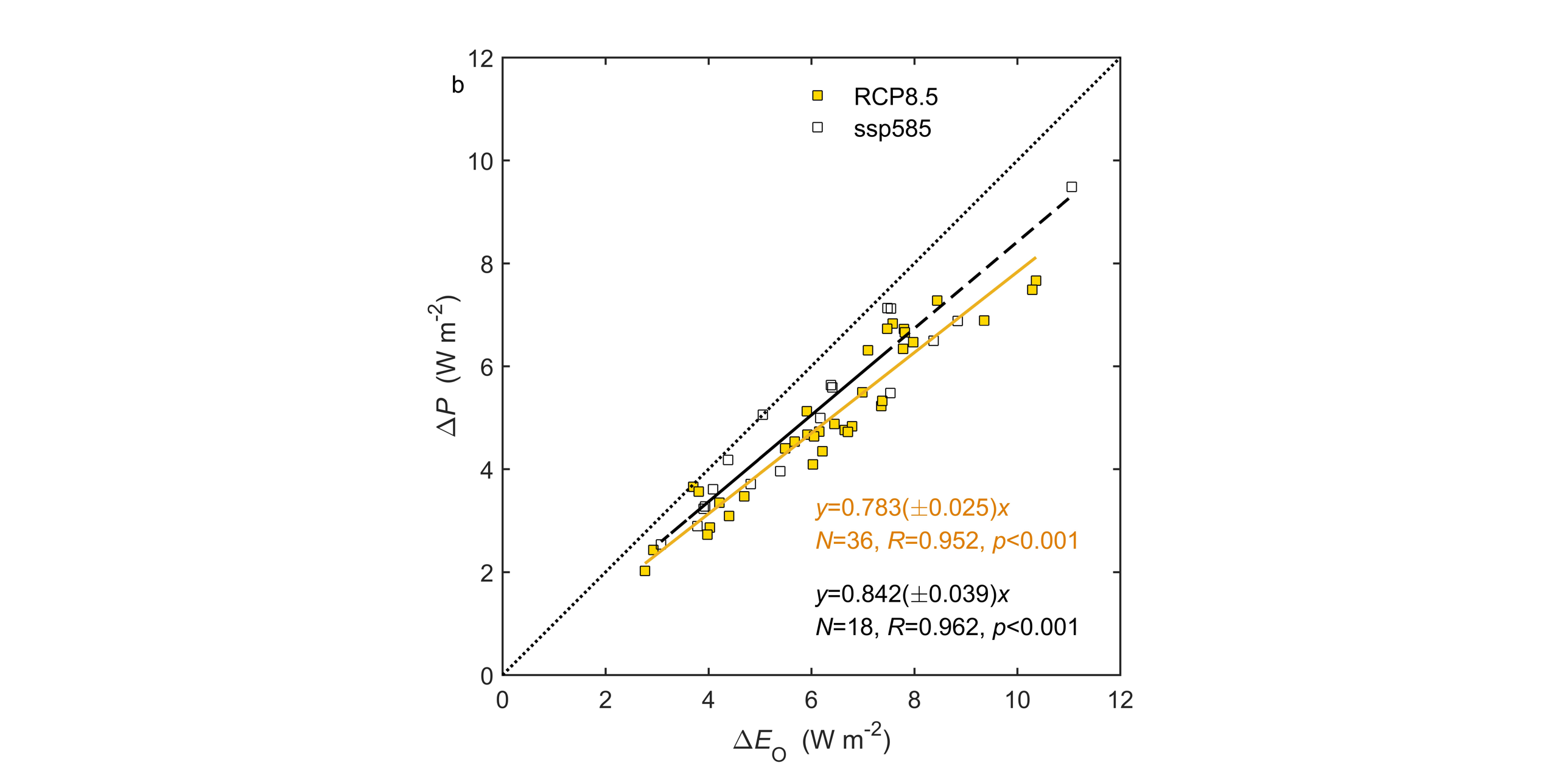


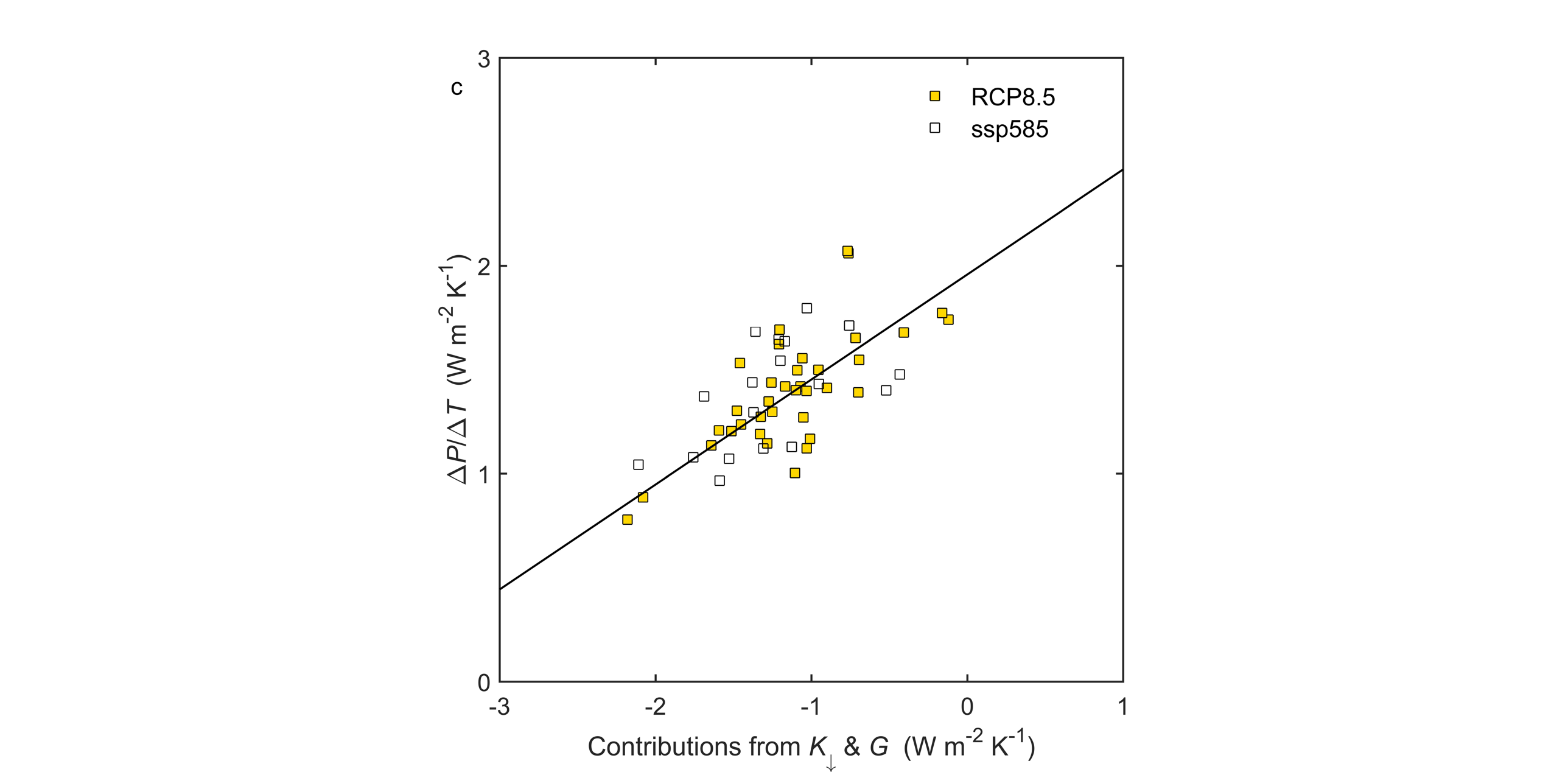


**Supplementary Figure 6. Dependence of precipitation temperature sensitivity (Δ*P*/Δ*T*) on feedback strength.** Here, the feedback strength *α* is approximated by the negative value of temperature sensitivity of ocean heat storage (-Δ*G*/Δ*T*) from a 4×CO_2_ simulation using the same models. The solid lines represent linear regression with the regression statistics noted (*R*, linear correlation), Description of model scenarios is given in Figure 1 caption.


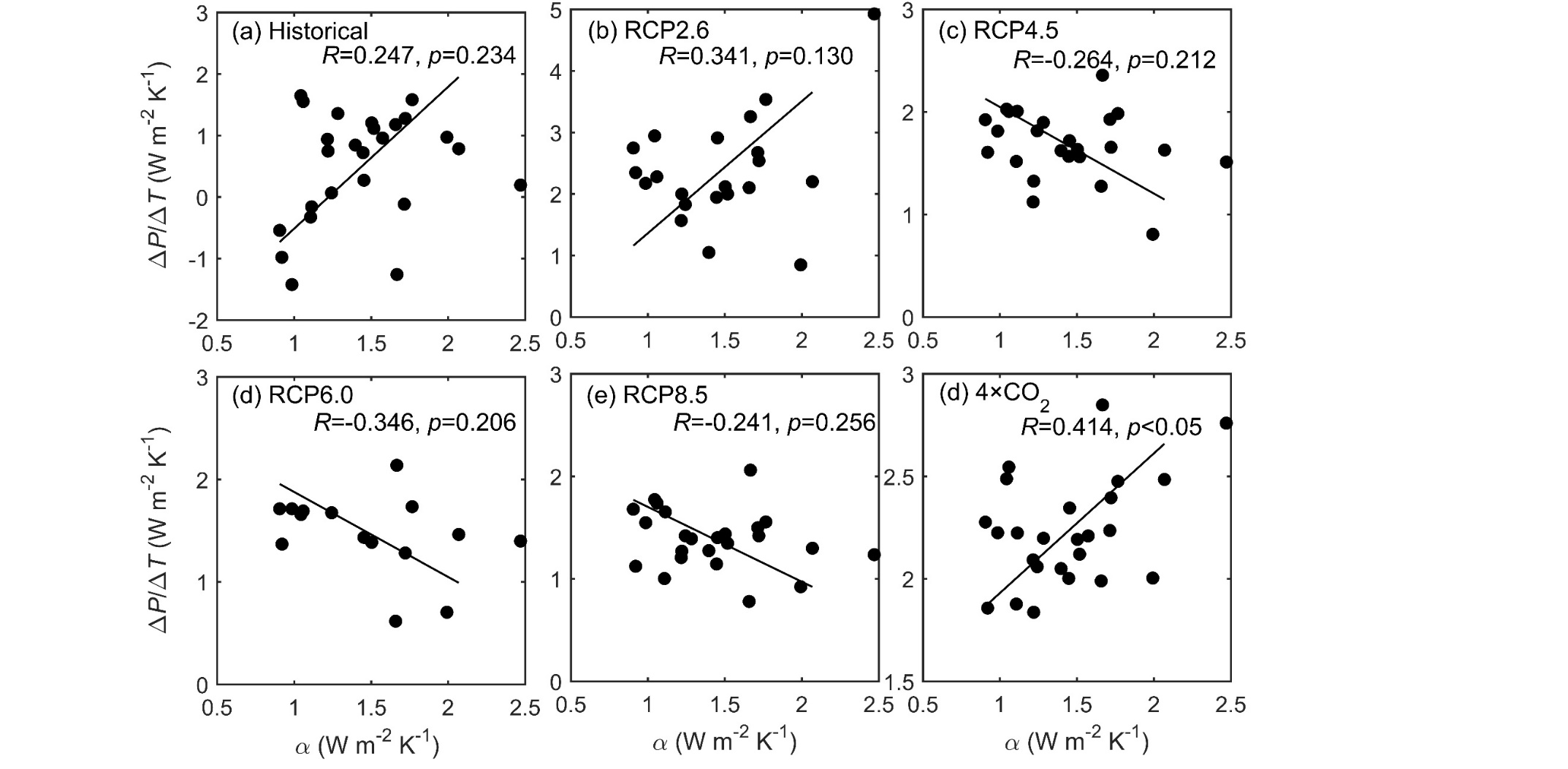


**Supplementary Figure 7. Relationship between ocean albedo and ocean incoming shortwave radiation at high latitudes (north of 60^o^ N and south of 60^o^ S).** **a**, annual mean ocean albedo *a* versus annual mean incoming shortwave radiation *K*_↓_ according to the CERES observation (<https://ceres.larc.nasa.gov/data/>). **b**, inter-model spread in the *a* and *K*_↓_ temperature sensitivities for CMIP5 historical simulations. The solid lines represent linear regression with the regression statistics noted (*N*, number of year in panel a and model experiments in panel b; *R*, linear correlation).


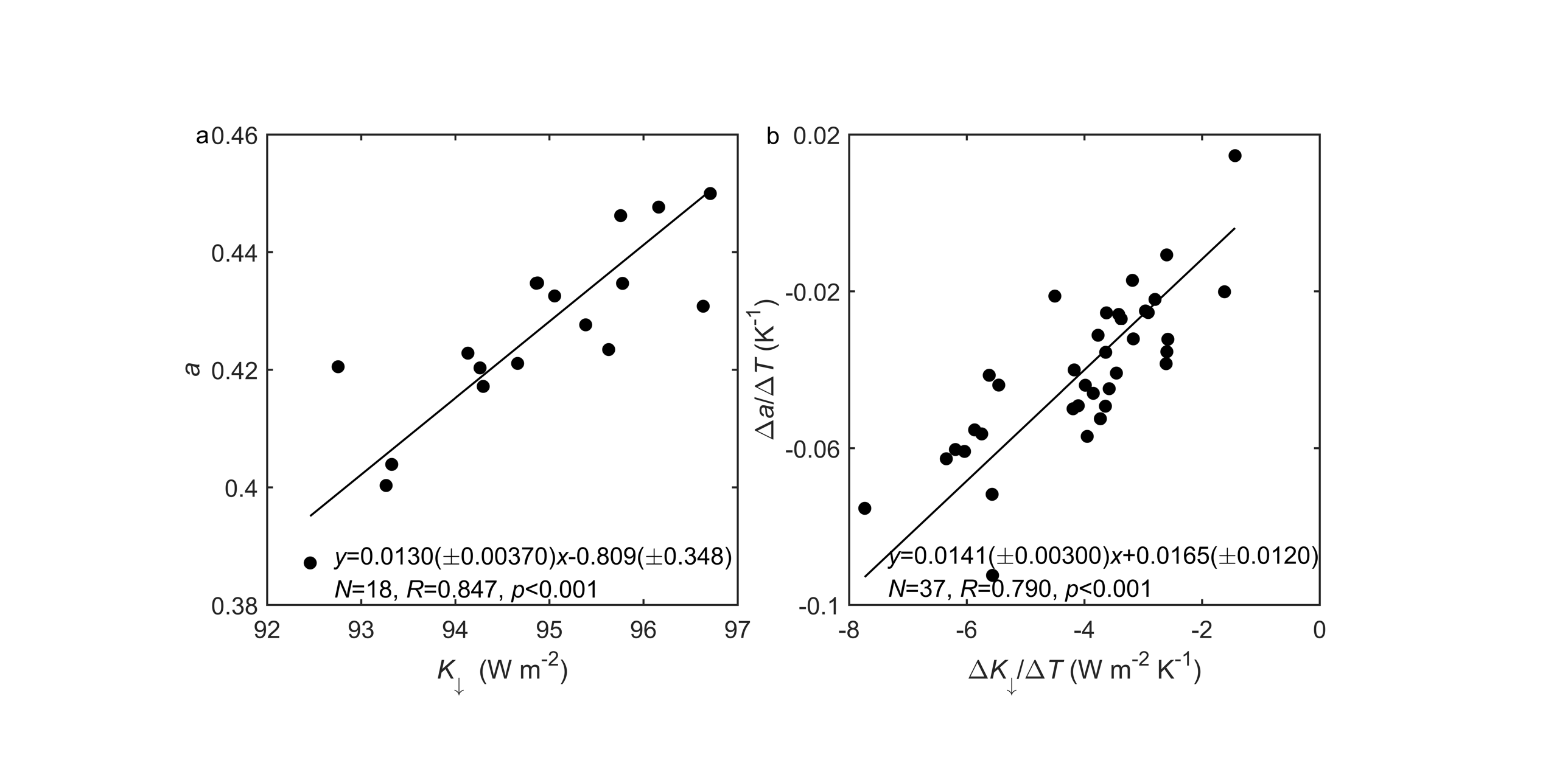


**Supplementary Figure 8. Comparison of different definitions of the hydrological climate sensitivity.** Data points are annual mean values from the IPSL-CM5A-LR model simulations for three climate scenarios. Solid red circles denote the first and last 10 years of the 4×CO_2_ simulation. Red pluses denote the 10-year mean values. In the present study, the slope of *P* versus *T* is approximated by precipitation temperature sensitivity Δ*P*/Δ*T*.


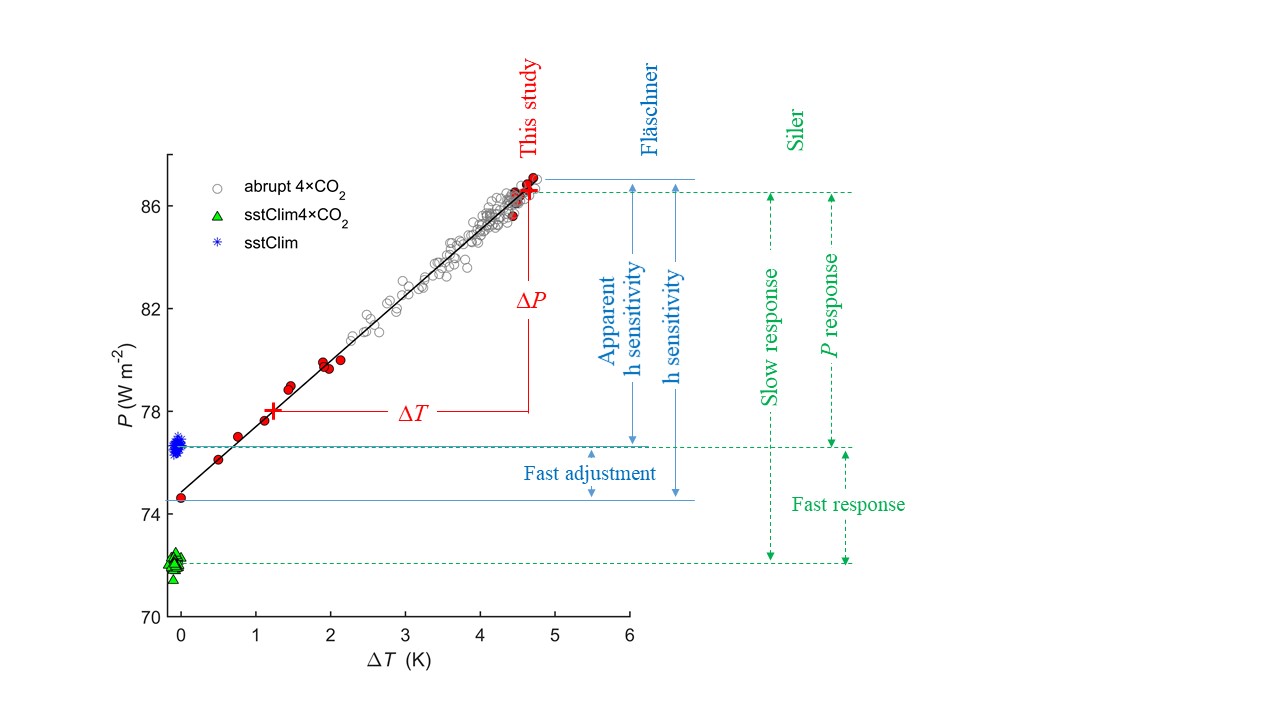


**Supplementary Fig. 9.** **Comparison of regional and global analysis using CMIP5 historical simulations.** **a**, Component contributions to global precipitation temperature sensitivity Δ*P*/Δ*T* calculated with Equation (1) using global mean values as inputs. **b**, Component contributions from a regional diagnostic analysis, where Equation (1) was applied separately to polar (north 60^o^ N and south of 60^o^ S) and non-polar grids (between 60^o^ N and of 60^o^ S), and the result was weighted by the area fraction of each group to give the global mean value. Red: sum of the five component contributions; yellow: contribution by Bowen ratio change; blue: contribution by surface albedo change; light blue: contribution by change in surface downward shortwave radiation; magenta: contribution by change in surface net longwave radiation; grey: contribution by change in ocean heat storage. Error bars are ± one standard deviation.


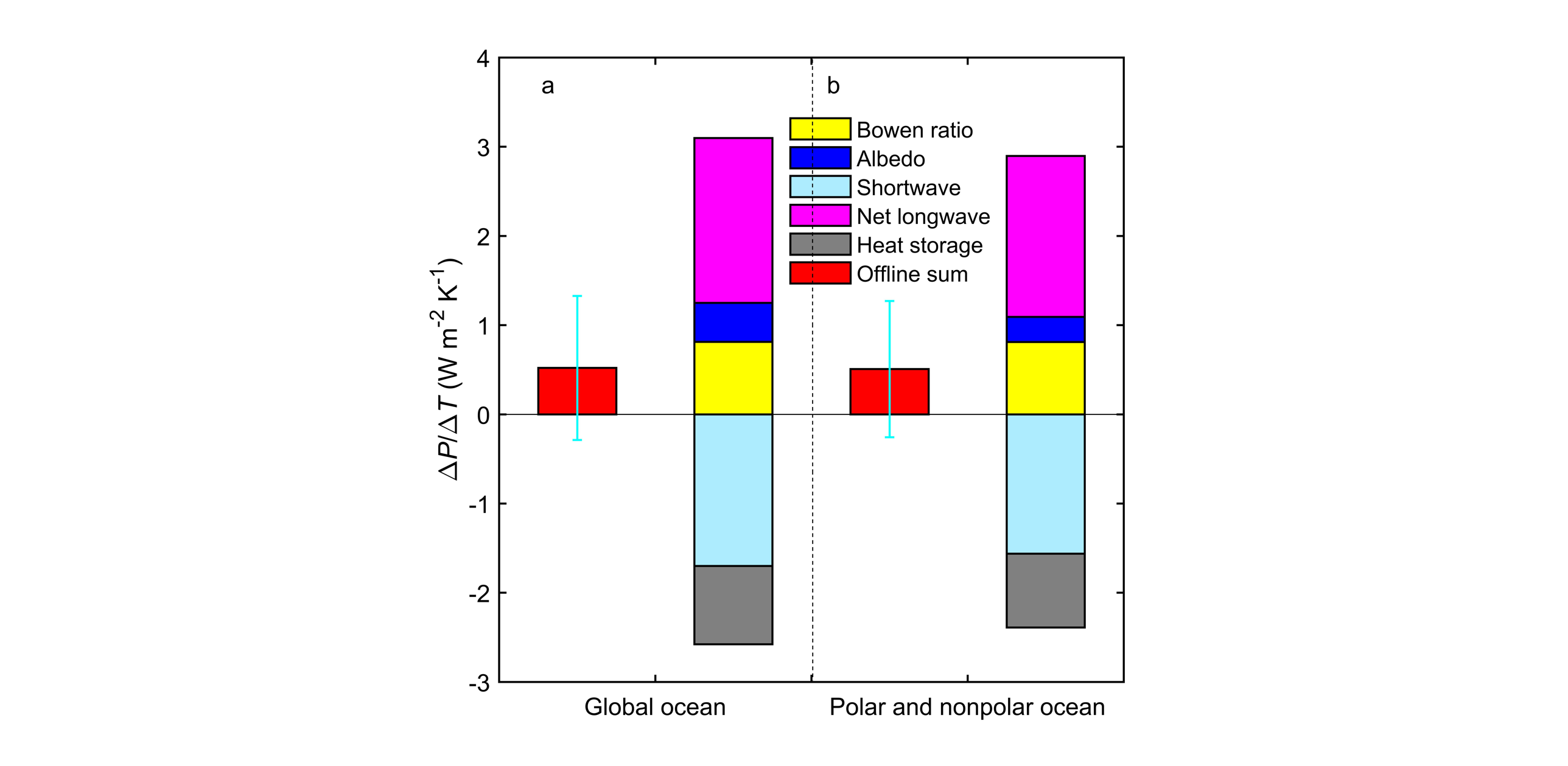


**Supplementary Table 1. Regression of changes in global precipitation and in ocean evaporation.** For each CMIP scenario, *s* is the slope of linear regression between changes in global precipitation (Δ*P*) and ocean evaporation (Δ*E*_O_) across models (with intercept forced through zero), where Δ*P* and Δ*E*_O_ are differences in global precipitation and ocean evaporation, respectively, between the last and the first 10-years of each model simulation. For MERRA-2, *s* is the slope of linear regression between annual global *P* and global *E*_o_ (with intercept forced through zero). Uncertainty range is ± one standard deviation, estimated as half of the 95% confidence bound on the regression slope. *N* – number of models (climate scenarios) or number of years (reanalysis); *φ*－land modifier, the ratio of land evaporation change to ocean evaporation change; *R*－linear regression coefficient. All correlations are significant at *p* < 0.001.

|  | ***N*** | ***s*** | ***φ*** | ***R*** |
| --- | --- | --- | --- | --- |
| **Climate model scenario** | | | | |
| CMIP5 Historical | 36 | 0.754±0.054 | 0.152±0.185 | 0.987 |
| CMIP5 RCP2.6 | 25 | 0.822±0.027 | 0.385±0.094 | 0.986 |
| CMIP5 RCP4.5 | 34 | 0.821±0.027 | 0.381±0.093 | 0.966 |
| CMIP5 RCP6.0 | 19 | 0.825±0.047 | 0.395±0.163 | 0.946 |
| CMIP5 RCP8.5 | 37 | 0.783±0.025 | 0.253±0.085 | 0.952 |
| CMIP5 4×CO2 | 25 | 0.762±0.013 | 0.180±0.046 | 0.984 |
| CMIP6 ssp585 | 18 | 0.842±0.039 | 0.455±0.136 | 0.962 |
| **Reanalysis** | | | | |
| MERRA-2 | 38 | 0.695±0.013 | -0.051±0.045 | 0.992 |

**Supplementary Table 2. Empirical constraints on the energy balance components at the ocean surface.** Refer to Figure S3 for symbol definitions.

| **Variable** | **Mean** | **S.D.** | **Reference or data source** |
| --- | --- | --- | --- |
| **Temperature sensitivity** | | | |
| $\frac{\Delta\beta}{\Delta T}$ (K^-1^) | -0.00834 | 0.000188 | Yang & Roderick (ref. 2) |
| $\frac{\Delta a}{\Delta T}$ (K^-1^) | -0.00653 | 0.00147 | CERES; Kato et al (ref. 3) |
| $\frac{\Delta K_{\downarrow}}{\Delta T}$ (W m^-2^ K^-1^) | -2.93 | 0.276 | Reanalysis products |
| $\frac{\Delta L_{\downarrow}}{\Delta T}$ (W m^-2^ K^-1^) | 7.51 | 0.672 | Reanalysis products |
| $\frac{\Delta L_{\uparrow}}{\Delta T}$ (W m^-2^ K^-1^) | 5.24 | 0.0191 | Stefan-Boltzmann Law |
| $\frac{\Delta G}{\Delta T}$ (W m^-2^ K^-1^) | 0.625 | 0.0257 | Cheng et al. (ref. 4) |
| **Ocean energy balance components** | | | |
| *R*_n_-*G* (W m^-2^) | 116 |  | Wild et al. (ref. 5) |
| *K_↓_* (W m^-2^) | 185 |  |  |
| *a* | 0.0811 |  |  |
| *β* | 0.160 |  |  |

**Supplementary Table 3. Sensitivity of incoming surface shortwave *K*_↓_ and longwave radiation *L*_↓_** **to global temperature *T*.** The sensitivity value is calculated as the regression slope of the annual mean *K*_↓_ or *L*_↓_ over ocean grids against the global mean temperature and adjusted slightly to remove the bias in *K*_↓_ or *L*_↓_ in reference to the CERES value (<https://ceres.larc.nasa.gov/data/>). Also shown is the coefficient of determination R^2^. All regressions are significant at *p* < 0.0001. The MERRA-2 Δ*K*_↓_/Δ*T* (value in parentheses) is excluded from the mean value given in Supplementary Table 2.

|  | Δ*K*_↓_/Δ*T* | |  | Δ*L*_↓_/Δ*T* | |  |
| --- | --- | --- | --- | --- | --- | --- |
| Data source | W m^-2^ K^-1^ | R^2^ |  | W m^-2^ K^-1^ | R^2^ | Period |
| NOAA-CIRES  <https://psl.noaa.gov/> | -2.36 | 0.27 |  | 6.28 | 0.92 | 1851-2014 |
| NCEP-NCAR  <https://psl.noaa.gov/> | -3.43 | 0.63 |  | 8.53 | 0.96 | 1948-2019 |
| JRA-55  <https://rda.ucar.edu/> | -3.55 | 0.63 |  | 6.17 | 0.95 | 1958-2013 |
| ERA-5  <https://cds.climate.copernicus.eu/> | -2.41 | 0.52 |  | 5.54 | 0.95 | 1980-2019 |
| MERRA-2  <https://esgf-node.llnl.gov/search/create-ip/> | (-9.16) | 0.52 |  | 8.90 | 0.89 | 1980-2019 |

**Supplementary Table 4. List of CMIP5 and CMIP6 climate model simulations used in this study.** The CMIP5 simulation periods for historical, future (RCP2.6, RCP 4.5, RCP6.0 and RCP8.5) and 4×CO2 scenarios are 1850 - 2005, 2006 - 2100 and 1850 - 1999, respectively. For CMIP6 ssp5-8.5, the simulation period is 2015-2100. Symbol T denotes availability of tree fraction data. Model CMCC-CMS does not output evaporation data and is not used in Fig. 1. BNU-ESM historical experiment is not used for tree fraction analysis presented in Supplementary Fig. 2 because it shows an unrealistically large tree fraction increase in the historical period. Model GISS-E2-R RCP2.6 has an unusually high global precipitation temperature sensitivity. Unless stated otherwise, GISS-E2-R RCP2.6 is excluded from the analysis.

|  | **Model name** | **Historical** | **RCP2.6** | **RCP4.5** | **RCP6.0** | **RCP8.5** | **4×CO_2_** | | **ssp585** |
| --- | --- | --- | --- | --- | --- | --- | --- | --- | --- |
| **CMIP5** | | | | | | | | | |
|  | ACCESS1.0 | Y | N | Y | N | Y | Y |  | |
|  | ACCESS1.3 | Y | N | Y | N | Y | Y |  | |
|  | BCC-CSM1.1 | Y | Y | Y | Y | Y | Y |  | |
|  | BCC-CSM1.1m | Y | Y | Y | Y | Y | Y |  | |
|  | BNU-ESM | Y, T | Y, T | Y, T | N | Y, T | Y, T |  | |
|  | CCSM4 | Y | Y | Y | Y | Y | Y |  | |
|  | CESM1(CAM5) | Y | Y | Y | Y | Y | N |  | |
|  | CMCC-CESM | Y, T | N | N | N | Y, T | N |  | |
|  | CMCC-CM | Y | N | Y | N | Y | N |  | |
|  | CMCC-CMS | Y | N | Y | N | Y | N |  | |
|  | CNRM-CM5 | Y | Y | Y | N | Y | Y |  | |
|  | CSIRO-Mk-3-6-0 | Y | Y | Y | Y | Y | Y |  | |
|  | CanESM2 | Y | Y | Y | N | Y | Y |  | |
|  | FGOALS-g2 | Y | Y | Y | N | Y | Y |  | |
|  | GFDL-CM3 | Y, T | Y, T | Y, T | Y, T | Y, T | Y, T |  | |
|  | GFDL-ESM2G | Y, T | Y, T | Y, T | Y, T | Y, T | Y, T |  | |
|  | GFDL-ESM2M | Y, T | Y, T | Y, T | Y, T | Y, T | Y, T |  | |
|  | GISS-E2-H | Y | Y | Y | Y | Y | Y |  | |
|  | GISS-E2-H-CC | Y | N | Y | N | Y | N |  | |
|  | GISS-E2-R | Y | Y | Y | Y | Y | Y |  | |
|  | GISS-E2-R-CC | Y | N | Y | N | Y | N |  | |
|  | HadGEM2-CC | Y, T | N | Y, T | N | Y, T | N |  | |
|  | HadGEM2-ES | Y | Y, T | Y, T | Y, T | Y, T | Y, T |  | |
|  | INM-CM4 | Y, T | N | Y, T | N | Y, T | N |  | |
|  | IPSL-CM5A-LR | Y, T | Y, T | Y, T | Y, T | Y, T | Y, T |  | |
|  | IPSL-CM5A-MR | Y, T | Y, T | Y, T | Y, T | Y, T | Y, T |  | |
|  | IPSL-CM5B-LR | Y, T | NT | Y, T | N | Y, T | Y, T |  | |
|  | MIROC-ESM | Y, T | Y, T | Y, T | Y, T | Y, T | Y, T |  | |
|  | MIROC-ESM-CHEM | Y, T | Y, T | Y, T | Y, T | Y, T | NT |  | |
|  | MIROC5 | Y, T | Y, T | Y, T | Y, T | Y, T | N |  | |
|  | MPI-ESM-LR | Y, T | Y, T | Y, T | N | Y, T | Y, T |  | |
|  | MPI-ESM-MR | Y, T | Y, T | Y, T | N | Y, T | Y, T |  | |
|  | MPI-ESM-P | Y, T | N | N | N | N | Y, T |  | |
|  | MRI-CGCM3 | Y | Y | Y | Y | Y | Y |  | |
|  | MRI-ESM1 | Y | N | N | N | Y | N |  | |
|  | NorESM1-M | Y | Y | Y | Y | Y | Y |  | |
|  | NorESM1-ME | Y | Y | Y | Y | Y | N |  | |
| **CMIP6** | | | | | | | | | |
|  | AWI-CM-1-1-MR |  |  |  |  |  |  | Y | |
|  | BCC-CSM2-MR |  |  |  |  |  |  | Y | |
|  | CAMS-CSM1-0 |  |  |  |  |  |  | Y | |
|  | CanESM5 |  |  |  |  |  |  | Y | |
|  | CESM2 |  |  |  |  |  |  | Y | |
|  | CESM2-WACCM |  |  |  |  |  |  | Y | |
|  | EC-Earth3 |  |  |  |  |  |  | Y | |
|  | EC-Earth3-Veg |  |  |  |  |  |  | Y | |
|  | FGOALS-f3-L |  |  |  |  |  |  | Y | |
|  | FGOALS-g3 |  |  |  |  |  |  | Y | |
|  | GFDL-ESM4 |  |  |  |  |  |  | Y | |
|  | INM-CM4-8 |  |  |  |  |  |  | Y | |
|  | INM-CM5-0 |  |  |  |  |  |  | Y | |
|  | IPSL-CM6A-LR |  |  |  |  |  |  | Y | |
|  | MIROC6 |  |  |  |  |  |  | Y | |
|  | MPI-ESM1-2-HR |  |  |  |  |  |  | Y | |
|  | MRI-ESM2-0 |  |  |  |  |  |  | Y | |
|  | NESM3 |  |  |  |  |  |  | Y | |

**Supplementary References**

1. Pendergrass, A. G. & Hartmann, D. L. The atmospheric energy constraint on global-mean precipitation change. *J. Clim*. **27**, 757–768 (2014).
2. Yang, Y. & Roderick, M. L. Radiation, surface temperature and evaporation over wet surfaces. *Q. J. R. Meteorol. Soc*. **145**, 1118–1129 (2019).
3. Kato, S. et al. Surface irradiances of edition 4.0 clouds and the Earth’s radiant energy system (CERES) energy balanced and filled (EBAF) data product. *J. Clim.* **31**, 4501–4527 (2018).
4. Cheng, L. et al. How fast are the oceans warming? *Science* **363**, 128–129 (2019).
5. Wild, M. et al. The energy balance over land and oceans: an assessment based on direct observations and CMIP5 climate models. *Clim. Dynam* **44**, 3393–3429 (2015).
